# A survey of surface imaging use in radiation oncology in the United States

**DOI:** 10.1002/acm2.12762

**Published:** 2019-11-19

**Authors:** Laura Padilla, Amanda Havnen‐Smith, Laura Cerviño, Hania A. Al‐Hallaq

**Affiliations:** ^1^ Department of Radiation Oncology Virginia Commonwealth University Richmond VA USA; ^2^ Department of Radiation Oncology Northfield Mayo Clinic Northfield MN USA; ^3^ Department of Medical Physics Memorial Sloan Kettering Cancer Center New York NY USA; ^4^ Department of Radiation and Cellular Oncology The University of Chicago Chicago IL USA

**Keywords:** Image‐guided radiotherapy, radiation oncology, surface‐guided radiotherapy, surface imaging, survey

## Abstract

Surface imaging (SI) has been rapidly integrated into radiotherapy clinics across the country without specific guidelines and recommendations on its commissioning and use aside from vendor‐provided information. A survey was created under the auspices of AAPM TG‐302 to assess the current status of SI to identify if there is need for formal guidance. The survey was designed to determine the institutional setting of responders, availability and length of its use, commissioning procedures, and clinical applications. This survey was created in REDCap, and approved as IRB exempt to collect anonymized data. Questions were reviewed by multiple physicists to ensure concept validity and piloted by a small group of independent physicists to ensure process validity. All full members of AAPM self‐identified as “therapy” or “other” were sent the survey link by email. The survey was active from February to March 2018. Of 3677 members successfully contacted, 439 completed responses; the summary of these responses provides insight on current surface imaging clinical practices, though they should not be assumed to be representative of radiation oncology as a whole. Results showed that 53.3% of respondents have SI in their clinics, mostly in treatment rooms, rarely in simulation rooms. Half of those without SI plan on purchasing it within 3 years. Over 10% have SI but do not use it clinically, 36.8% classify themselves as “expert” users, and 85.5% agreed/strongly agreed that SI guidelines are needed. Initial positioning with SI is most common for breast/chestwall and SRS/SBRT treatments, least common for pediatrics. Use of SI for intra‐fraction monitoring follows a similar distribution. Gating with SI is most prevalent for breast/chestwall (66.0%) but also used in SBRT (33.0%), and non‐SBRT lung/abdomen (<30%) treatments. SI is a rapidly growing technology in the field with widespread use for several anatomic sites. Guidelines and recommendations on commissioning and clinical use are warranted.

## 1 INTRODUCTION

Surface‐guided radiotherapy (SGRT) describes the integration of recent surface imaging (SI) technology into radiotherapy treatments. Surface imaging systems can be used as an aid for initial patient positioning, intra‐fraction monitoring, and in some cases, even respiratory motion management.^1^ These systems have been adopted in an increasing number of clinics over the past decade due to their ability to perform these tasks without the use of ionizing radiation. The technical characteristics and a description of how current commercially available surface imaging systems work have been described elsewhere.^2^ In brief, these systems can monitor the patient’s position in real time using optical light and compare it to a given reference from either the external contour of the planning CT or an SI system‐acquired capture. Typical applications of these systems, based on current literature, mainly include open‐mask stereotactic radiosurgery (SRS) procedures and breast radiotherapy, particularly deep inspiration breath‐hold (DIBH) treatments for left‐sided breast patients.^1^, ^3^ Literature describing SI use for other sites is more limited. While it is evident that this technology is being increasingly used in radiation oncology, its prevalence, implementation workflows, or scope of use in the field have not been described to date. An electronic survey was conducted in an effort to compile this information.

## 2 METHODS

A questionnaire was designed to assess the extent of use of SI for radiotherapy in the United States and gain more insight on its implementation in the field. Questions were crafted to inquire about the availability of this technology in clinics, existing commissioning procedures, and its role in current clinical practice regarding both its applications and common treatment sites of use (see Table S1). This survey was deemed IRB exempt after institutional board review at The University of Chicago as all the responses were anonymized and aggregated and could not be related back to the participants. The survey, along with text outlining its purpose, length, participation consent, and anonymity of results, was sent out via email to all full members of the American Association of Physicists in Medicine who self‐identified as specializing in “therapy” or “other” and had a mailing address in the U.S. Both the survey questions and the text used in the survey invitation are listed in Table S1. The survey was active from February to March of 2018.

Study data were collected and managed using REDCap (Research Electronic Data Capture) electronic data capture tools hosted at The University of Chicago.^4^ REDCap is a secure, web‐based application designed to support data capture for research studies, providing (a) an intuitive interface for validated data entry; (b) audit trails for tracking data manipulation and export procedures; (c) automated export procedures for seamless data downloads to common statistical packages; and (d) procedures for importing data from external sources.

Survey questions were organized into two sections. The first one was to determine the institutional setting of the responder, the availability and duration of use of the technology, and the commissioning process performed upon initial acquisition of the system(s). The second section focused on the clinical uses of surface imaging, including applications (e.g., initial positioning, intra‐fraction monitoring, gating) and types of treatment (e.g., anatomical site and type of procedure — conventional, stereotactic, pediatric). All questions were reviewed by more than ten physicists for concept validity. The survey was tested by a small cohort of physicists independent from the survey creators to ensure response process validity prior to deployment for data collection. The survey length was intended to be brief: 10 min for participants who had surface imaging and 2 min for those who did not.

## 3 RESULTS

There were 205 undeliverable emails of the 3882 emails originally sent. We received 509 responses, 439 were complete. Only complete responses were used. The overall response rate was 13.8% (from self‐identified “therapy” and “other” AAPM members). The response rate from “therapy” only members was 14.7%.

Table 1 summarizes the institutional setting of the respondents, prevalence of SI, and general information about its clinical implementation. Respondents with proton and photon treatment machines at their centers were given the choice to answer the questions for either photons, protons, or both. Five respondents chose to answer exclusively based on the use of SI for proton treatments. Due to this small number, these answers were combined with the photon treatment responses for clinical use and are not reported independently.

**Table 1:** Summary of respondent characteristics and general surface imaging (SI) information about prevalence and clinical implementation.

Respondent characteristics and prevalence of SI (n = 439)		
	n	%
Institutional setting		
Academic hospital	102	23.2
w/SI	74/102	72.5
Private/community practice	307	69.9
w/SI	152/307	49.5
Government‐owned center	14	3.2
w/SI	3/14	21.4
Other (including consulting)	16	3.6
w/SI	5/16	31.3
Solo physicist		
Yes	107	24.4
w/SI	37/107	34.6
No	332	75.6
w/SI	197/332	59.3
SI equipment in clinic		
Yes	234	53.3
No	205	46.7
No, but expected in 1 year	51/205	24.9
No, but expected in 3 years	49/205	23.9
No purchase plans	105/205	51.2

General information about SI in clinics (n = 234)		
	n	%
Year of installation		
prior to 2012	35	15.0
2012–2014	58	24.8
2015–present	139	59.4
Don't know	2	0.9
Vendor		
Single	196	83.8
Single, from vendor A	83/196	42.3
Single, from vendor B	90/196	45.9
Single, from vendor C	22/196	11.2
Single, from vendor D	0/196	0
Multiple (2)	34	14.5
Multiple (3)	4	1.7
Location in clinic		
Simulator	78	33.6^a^
SI used for gating at simulator	27/78	34.6
Treatment — photons	228	98.7^b^
MMI used to gate photon treatment	137/228	60.1
Treatment — protons	11	42.3^c^
MMI used to gate proton treatment	1/11	9.1
Clinical implementation		
Within a year	174	74.4
After 1–2 years	21	9.0
>3 years	8	3.4
Don't know	6	2.6
Not used clinically	25	10.7
References used for commissioning		
AAPM online — only	1	0.0
TG147 — only	4	1.7
Vendor's guidelines — only	86	36.8
Publications — only	0	0
Colleagues — only	4	1.7
Nothing	2	0.9
Don't know	22	9.4
More than one reference used	115	49.1
End‐to‐end test performed		
Yes	173	73.9
No	28	12.0
Don't know	33	14.1
Agreement on necessity for SI guidelines		
Strongly agree	98	41.9
Agree	102	43.6
Neither	23	9.8
Disagree	10	4.3
Strongly disagree	1	0.0
Expertise level for clinical users (n = 209)		
Expert	77	36.8
Developing	114	54.5
Amateur	12	5.7
Inexperienced	6	2.9

Abbreviations: MMI, Motion Management Interface; AAPM, American Association of Physicists in Medicine; TG, Task Group.

^a^ 232 respondents with SI have simulators in their clinics.

^b^ 231 respondents with SI have photon treatment machines in their clinic.

^c^ 26 respondents with SI have proton treatment machines in their clinic.

Over half of the respondents reported having SI systems in their clinic (53.3%). Of those who do not currently have it, 48.8% reported that their clinic plans to purchase an SI system within the next 1–3 years. Out of the respondents with SI in their clinics, most (59.4%) report their SI equipment was installed on or after 2015, and 10.7% indicated that although they have SI at their facilities, it is not being used clinically. Only 36.8% of reported users classify their level of expertise as “expert,” and 85.5% of all respondents with SI agree to strongly agree that guidelines for the clinical use of surface imaging are necessary.

### 3.1 Surface imaging for initial positioning

**Figure 1:**
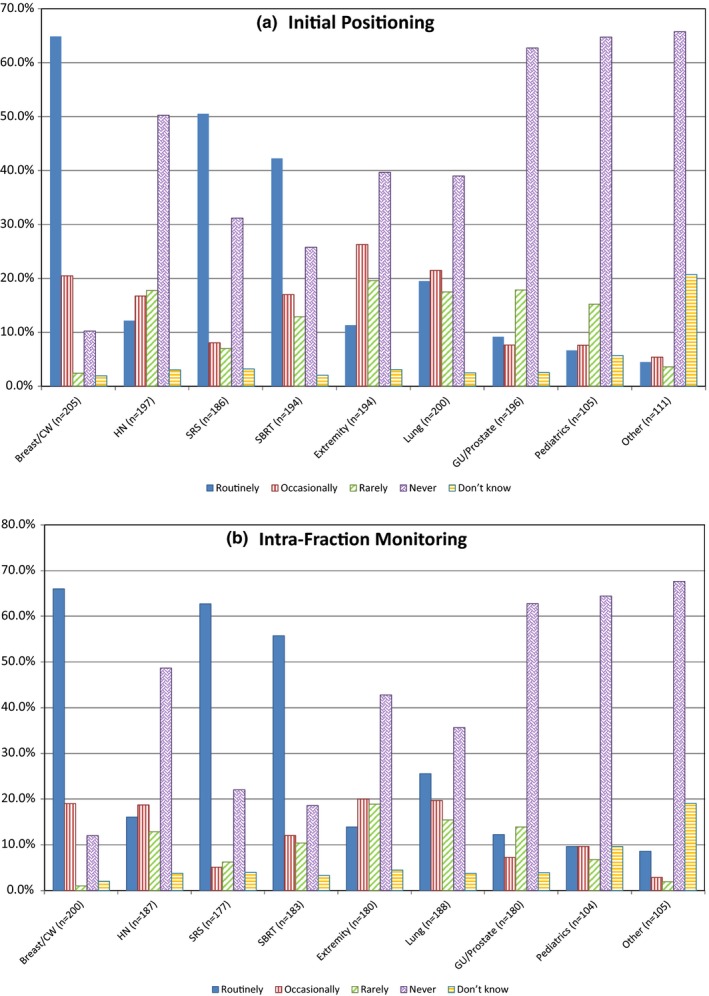
Use of surface imaging for initial patient positioning (a) and intra‐fraction monitoring (b) by site/treatment type. “Other” includes abdominal treatments (liver, pancreas, etc.), non‐GU/prostate pelvis treatments, primary brain, and electron treatments. Note the “n” for each site/treatment type is listed in the x‐axis. This number differs from 209 (total number of respondents using SI clinically) because some of them indicated these categories as “NA – Not Applicable.” NA responses have been excluded from these results.

Participants who indicated that SI has been clinically implemented in their department (n = 209) were asked to elaborate on their use of SI for initial positioning. This included specifying both the type of reference surface being used for this purpose (DICOM surface from the planning CT acquired during simulation, camera‐acquired surface at simulation, or camera‐acquired in the treatment room) and the treatments and sites for which SI was being employed for initial setup. Survey responses showed that the majority of users, 63.2%, perform initial positioning based on a single type of reference surface for every fraction of a patient’s treatment (DICOM surface). Only 7.4% of respondents use the camera‐acquired surface in the treatment room as their single reference for initial positioning. This number decreases to 1.0% when considering camera‐acquired surfaces at simulation. Almost 20% of users indicate that the type of reference surface used throughout a treatment course for initial positioning varies depending on treatment site or patient. The results showing the prevalence of SI for initial positioning per treatment site/type are compiled in [Fig. 1(a)]. The frequency of use of SI for initial positioning is highest for breast (routinely: 64.9%), SRS (routinely: 50.5%), and SBRT (routinely: 42.3%). It is rarely used for pediatric patients (never: 64.8%), GU/Prostate (never: 62.8%), and other pelvic or abdominal treatments (never: 65.8%). Note that respondents were given the option of selecting “Not Applicable” for treatment sites/types that are not treated in their clinic (see Table S1). Thus, the percentages shown in all figures are calculated based only on the respondents who perform those types of treatments in their clinics. The numbers of respondents included in each treatment site/type are indicated in parenthesis in each figure.

The survey also inquired about frequency of verification of the surface imaging position with internal imaging for the same treatments listed in Fig. 1. For both stereotactic radiosurgery (SRS) and stereotactic body radiotherapy (SBRT), almost all respondents using SI for initial positioning verify the position with internal imaging daily (93.5% and 92.2%, respectively). When used for lung and GU/prostate treatments, most users (76.1% and 67.6%, respectively) verify it daily, while for breast and extremities, internal imaging verification is split between daily (48.9% and 47.3%) and weekly (46.1% and 40.2%). Pediatric cases have the lowest percentage of daily verification (41.9%), and the highest percentage of positioning the patient without ever using internal imaging for verification (6.5%).

**Figure 2:**
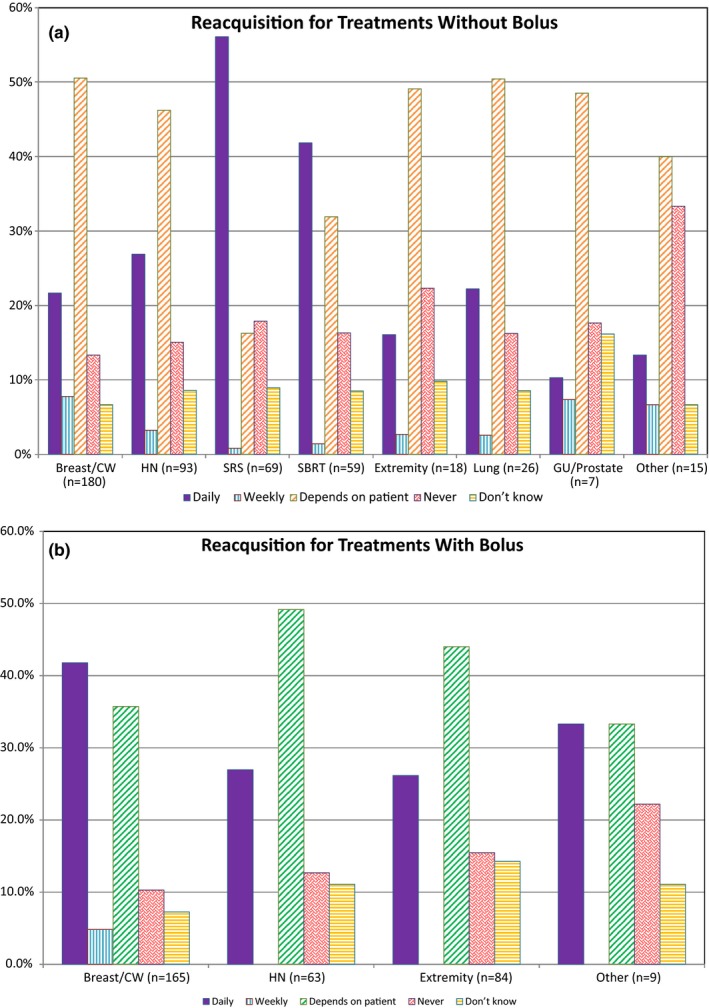
Frequency of reference surface reacquisition throughout the treatment course for different treatment sites/types separated by use of bolus. Non‐bolus treatments are depicted in (a), bolus treatments are shown in (b). Note the “n” for each site/treatment type is listed in the x‐axis. This number differs from the n in Fig. 1 because only respondents using SI for initial positioning of the indicated site/treatment type were given these questions. This number is further decreased in graph b of this figure because some respondents indicated that the use of bolus for these treatments is “NA — Not Applicable” in their clinic.

Since reference surface captures with in‐room cameras can be performed at any time during treatment, the frequency of reference surface acquisition was also investigated. Figure 2 shows the frequency of acquisition per treatment site divided into non‐bolus [Fig. 2(a)] and bolus [Fig. 2(b)] treatments. The frequency of capturing a new reference surface is higher for breast cancer treatments that require bolus than those that do not.

### 3.2 Surface imaging for intra‐fraction monitoring

Participants were also asked about their use of SI for intra‐fraction monitoring. As for initial positioning, both the type of reference surface utilized for intra‐fraction monitoring and the treatment sites/types where SI was used for this purpose were investigated. For intra‐fraction monitoring, results indicate that a single reference surface type is often used, with 41.4% of respondents using camera‐acquired surfaces in the treatment room and 22.2% using the DICOM surface. Almost 30% of respondents indicated that they select the reference surface type for intra‐fraction monitoring depending on the treatment site or patient. The breakdown of the use of SI for intra‐fraction monitoring per treatment site/type is shown [Fig. 1(b)]. Similar to initial positioning, the frequency of SI use for intra‐fraction monitoring is highest for breast (routinely: 66.0%), SRS (routinely: 62.7%), and SBRT (routinely: 55.7%), and lowest for pediatric patients (never: 64.4%), GU/Prostate (never: 62.8%), and other pelvic or abdominal treatments (never: 67.6%).

### 3.3 Surface imaging for respiratory gating

Lastly, the survey investigated the use of surface imaging for respiratory gating. Of the respondents with surface imaging available in the simulation room, over a third (34.6%) use it for respiratory gating. The reported use of SI for respiratory gating during treatment varied depending on the treatment site/type. Respondents who reported using SI for intra‐fraction motion monitoring were asked if they used this technology for respiratory motion management during treatment. Of those monitoring breast/chest wall patients, 80.2% use SI for respiratory motion management while 7% use a non‐SI system. Of those who treat SBRT, 48.3% use SI for respiratory motion management while 24.5% use a non‐SI system. Of those who treat non‐SBRT lung cancer, 49.1% use SI for respiratory motion management while 23.7% use a non‐SI system. Of those who treat abdominal cancers, 28.2% use SI for respiratory motion management while 30.2% use a non‐SI system. To put these numbers into perspective, 66.0% of all clinical SI users utilize this technology for respiratory motion management of breast and chest wall treatments, 33.0% use it for SBRT treatments, 26.8% use it for non‐SBRT lung, and 20.1% for abdomen non‐SBRT.

**Figure 3:**
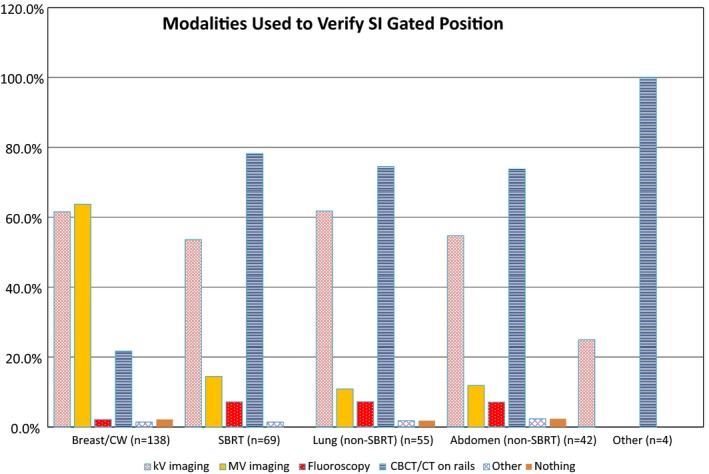
Internal imaging modalities used per treatment site/type to verify respiratory gating position with SI as reported by respondents using SI for respiratory gating during treatment.

Respondents were also asked what internal imaging verification tools, if any, they used to confirm the respiratory gating position given by SI. Figure 3 summarizes those results. Respondents were allowed to select more than one modality per site, if applicable. Except for breast/chest wall treatments, which are verified with planar MV imaging slightly more frequently than with planar kV imaging (63.8% and 61.6%, respectively), the most common modalities for verification are planar kV imaging and CBCT/CT on rails. Volumetric imaging (CBCT/CT on rails) is more widely used than planar kV imaging for SBRT (78.3% vs 53.6%), non‐SBRT lung (74.5% vs 61.8%), and non‐SBRT abdomen (73.8% vs 54.8%).

## 4 DISCUSSION

The use of SI in radiotherapy is increasing rapidly and it is important to understand how and for what purpose it is being used. This can help characterize the current status of the technology and identify areas of need for official guidelines and recommendations for safe application. To the authors’ knowledge, this is the first survey ever published on this topic. Although this survey has a low response rate, this limitation is not uncommon in such studies in medical physics.^5^, ^6^

Due to the anonymous nature of the survey, no specific information was collected on the respondents’ employers, and over 75% of the respondents indicated that they were not solo physicists. Thus, it is not possible to determine the breadth of radiation oncology practices across the United States represented by the results of this survey and duplicate responses from the same institution may have been submitted. Nevertheless, the data presented in this article illuminate the current applications of this technology, and possible areas in need of further guidance from the professional society to ensure safe and efficient use of SI in the field.

Over half of all the respondents currently have surface imaging in their clinic. This number is anticipated to increase rapidly in the upcoming years as almost half of those who do not currently have a system in their department are planning to purchase one within the next 1–3 yr. Although the number of sites with surface imaging capabilities seems to be growing quickly, a quarter of the respondents indicate either slow clinical implementation (12.4% take longer than 1 yr) or no clinical use of the equipment at all (10.7%). These results indicate that the advantages that surface imaging offers — patient positioning and constant intra‐fraction monitoring without additional dose to the patient — are attractive features to the clinic, but the slower clinical implementation reported could be due to the lack of guidance on how to successfully integrate this technology clinically. This assessment is reinforced by the fact that the majority of the participants with surface imaging (85.5%) agree/strongly agree with the need for national recommendations on the use of these systems.

A large proportion of respondents with SI report having the same vendor (see Table 1), which was the first vendor to offer this technology in the U.S. market. Responses also indicate that surface imaging is most commonly found in treatment vaults (98.7%) rather than simulation rooms (33.6%). Since surface imaging systems have the capability of gating the treatment beam based on the patient’s position being in or out of tolerance, participants were asked if this feature was available and clinically used. A total of 57.7% of respondents with surface imaging in their clinic, including photon and proton treatment machines, reported using the beam gating capability.

The results collected in this study show that surface imaging is most commonly used for breast (with and without breath‐hold) and SRS treatments, which is reflective of the current body of literature published on this technology 1, 3. In addition, these two sites are expected to have a robust surface‐to‐target positional correlation which makes them ideal candidates for SI use. Respondents who indicate the use of SI for initial positioning, typically also use it for intra‐fraction monitoring. As seen in Fig. 1, the trends of use for initial positioning and intra‐fraction monitoring are very similar, although the percentages vary slightly. From the data analysis, the use of surface imaging for SBRT is also considerable. This is unsurprising as SI allows for real‐time patient monitoring throughout treatment, while also allowing for the treatment to be interrupted if patient motion exceeds a preset tolerance when beam gating capabilities are enabled. This can improve the safety of treatment delivery, which is especially important for high‐risk treatments such as SBRT. Although for various sites, the patient’s surface may not be the most sensitive surrogate to indicate tumor motion during treatment, depending on the treatment site, it still allows for the detection of gross patient motion during radiation delivery.

Data show that DICOM surfaces are commonly selected as the reference surface for initial positioning, while camera‐acquired surfaces in the treatment room are used for intra‐fraction monitoring. This indicates that users generally rely on the DICOM surface for initial patient setup, which decreases the possibility of introducing systematic errors in positioning from using a reference surface different from that of the planning CT. The most common exceptions for which the reference surfaces utilized for initial positioning are routinely reacquired were SRS and SBRT treatments (56.1% and 41.8%, respectively), and breast and chest wall treatments using bolus (41.8%). SRS is by definition a single fraction treatment, although this term is now increasingly being used in the field to refer to hypofractionated stereotactic intracranial treatments as well. This could explain why some respondents indicated that the reference surface for initial positioning is routinely reacquired for SRS treatments. In the case of both SRS and SBRT, patient positioning with surface imaging is verified daily with internal imaging 95.0% and 93.5% of the time, according to the collected data. Therefore, any systematic errors the reference surface selection could introduce during initial setup are irrelevant as the treatment position is ultimately determined based on internal imaging. It is important to note that although the reference surface selection for initial positioning will not impact the treatment delivered when internal imaging is performed, it can still unnecessarily increase the setup time and internal imaging required for setup if the reference surface selected is inadequate.

For intra‐fraction monitoring, utilizing a camera‐acquired reference surface in the treatment room can make surface imaging systems more sensitive to patient motion. The use of the DICOM surface for intra‐fraction monitoring could decrease the effectiveness of motion detection due to the inevitable discrepancies between current and planned positions that are bound to arise on a daily basis. Utilizing an acquired reference, after confirmation with internal imaging, zeroes out these discrepancies and allows for any intra‐fraction motion to get detected by the system.

Although the frequency of use of SI for breath‐hold treatments is 66% for breast cases, less than a third of the respondents who use SI clinically use it for respiratory management for other sites such as non‐SBRT lung and abdomen, and SBRT. This could be due to other factors, including a lack of publications demonstrating surface imaging respiratory motion management for other sites, a lower percentage of respiratory motion management performed for those treatments across the field, or other practical considerations. One such consideration is that treatments necessitating acquisition of a CBCT under breath‐hold for accurate patient setup increase the likelihood of occlusion of the patient’s surface, not only by the gantry but also by the imaging arms. This in turn can lead to poor surface detection by SI systems thus jeopardizing the quality and consistency of the respiratory motion management during setup imaging.

The low rate of SI use in pediatric cases is unexpected as this technology could help better monitor these patients during treatment and reduce the imaging dose during setup. This could be due to the fact that the patient needs to be uncovered during treatment for SI monitoring, and some clinics might bypass its use in order to make the patient more comfortable. Also surprising is the low rate of SI utilization in head and neck, given its successful implementation for intracranial SRS. This could be related to the fact that many clinics use a closed‐face mask for immobilizing head and neck patients thus negating the utility of surface imaging.

## 5 CONCLUSIONS

Surface imaging is an attractive imaging technology due to its ability to aid in initial positioning, intra‐fraction monitoring, and beam gating without the use of ionizing radiation. Although our results cannot be generalized due to the limited response rate of the survey, they present the medical physics community with an overview of current uses and practices in the field. Currently, our results indicate that the majority of clinical applications are for breast (with and without DIBH), SRS, and SBRT treatments. Lower rates of use were reported for other treatments such as for pediatric and lung cancer. One‐quarter of respondents with SI capabilities reported no or slow clinical implementation. As the rates of adoption are expected to increase, and different techniques for commissioning and implementation may introduce systematic errors into patient setup and monitoring, national guidelines on the clinical implementation of surface imaging are needed to expedite and standardize its use.

## CONFLICT OF INTEREST

H. Al‐Hallaq reports royalties for computer‐aided diagnosis software for breast cancer detection, licensed from The University of Chicago, and grants from Varian Medical Systems, not related to this work. Laura Cerviño reports grants from Varian Medical Systems outside the scope of this work.

## Supporting information


**Table S1** **.** Survey questions and answer choices. Questions indicated with alphabetical designations are branches from a question with the corresponding numeral. For respondents with no surface imaging systems in their clinic, the survey ends after question 6b.
