# Supplementary material for: A survey of surface imaging use in radiation oncology in the United States
Source: J Appl Clin Med Phys. 2019 Nov 19;20(12):70–7. doi: 10.1002/acm2.12762 (PMC6909172; doi:10.1002/acm2.12762)
Supplement: Supplementary file 1 — Table S1 . Survey questions and answer choices. Questions indicated with alphabetical designations are branches from a question with the corresponding numeral. For respondents with no surface imaging systems in their clinic, the survey ends after question 6b. [file ACM2-20-70-s001.docx]

Appendix Table A. Survey questions and answer choices. Questions indicated with alphabetical designations are branches from a question with the corresponding numeral. For respondents with no surface imaging systems in their clinic, the survey ends after question 6b.

| **Num** | **Question** | **Answer Choices** | | | | | | |
| --- | --- | --- | --- | --- | --- | --- | --- | --- |
| **1** | Which of the following best describes your primary institutional setting? (All subsequent questions will apply to this institution.) | Academic hospital | Private/Community practice | Government-owned center | Other (includes consultant services) |  |  |  |
| **2** | Are you the solo physicist at this institution? | Yes | No |  |  |  |  |  |
| **3** | How many simulation units are at your institution(s)? (Enter zero if none. Include all sites at which you provide service.) | Fill in integer |  |  |  |  |  |  |
| **4** | How many photon treatment units are at your institution(s)? (Enter zero if none. Include all sites at which you provide service.) | Fill in integer |  |  |  |  |  |  |
| **5** | How many proton treatment rooms are at your institution(s)? (Enter zero if none. Include all sites at which you provide service.) | Fill in integer |  |  |  |  |  |  |
| **6** | Does your institution have at least 1 commercial surface imaging system (i.e., VisionRT, OSMS, C-RAD, HumediQ)? | Yes | No |  |  |  |  |  |
| **6a** | If "Yes," indicate the vendor of the commercial system at your institution (check all that apply). You cannot choose the "I don't know the vendor or it's not listed above" option if you have also chosen other | AlignRT | Varian OSMS | C-RAD | HumediQ | I don't know the vendor or it's not listed above. |  |  |
| **6b** | If "No," is your institution considering purchasing one? (*Survey ends here if no SI Systems at institution*). | Yes, within 1 year | Yes, within 3 years | No |  |  |  |  |
| **7** | When was the first surface imaging system installed at your institution? | Prior to 2012 | 2012-2014 | 2015-present | Don't know |  |  |  |
| **8** | What references did your institution use to commission and accept the surface imaging system? (Check all that apply.) | AAPM online presentation | AAPM TG-147 | Vendor's guidelines | Publications | Discussion with colleagues | Nothing | Don't know |
| **9** | Have you performed end-to-end testing (simulation-to-treatment) of your surface imaging system at least once? | Yes | No | Don't Know |  |  |  |  |
| **10** | Please select the statement that best describes your opinion: "National recommendations on the clinical applications of surface imaging would be beneficial to my clinical practice." | Strongly Agree | Agree | Neither Agree nor Disagree | Disagree | Strongly Disagree |  |  |
| **11** | Please indicate the number of simulators at your institution that have surface imaging capability. | Fill in integer |  |  |  |  |  |  |
| **11a** | Do you use surface imaging at the simulator for gating (4DCT, gated and/or breathhold scans)? | Yes | No |  |  |  |  |  |
| **12** | Please indicate the number of photon machines (i.e., LINACs, Tomotherapy, CyberKnife, ViewRay) at your institution that have surface imaging capability. | Fill in integer |  |  |  |  |  |  |
| **12a** | Does your surface imaging system have an interface with your photon treatment machine to automatically turn the beam off when patient positions exceed tolerance values? | Yes, on all machines | Yes, on some machines | Yes, but it's not used clinically | No | Don't Know |  |  |
| **13** | Please indicate the number of proton treatment rooms at your institution that have surface imaging capability. | Fill in integer |  |  |  |  |  |  |
| **13a** | Does your surface imaging system have an interface with your proton treatment machine to automatically turn the beam off when patient positions exceed tolerance values? | Yes, on all machines | Yes, on some machines | Yes, but it's not used clinically | No | Don't Know |  |  |
| **14** | You have indicated that surface imaging systems are available in both photon and proton treatment rooms at your institution. Please indicate if the remainder of your answers will reflect the clinical use of surface imaging at your institution for photon treatments only, proton treatments only, or both. | Photons | Protons | The clinical use of surface imaging is performed in the same way for both modalities |  |  |  |  |
| **15** | How long after installation did you start using surface imaging clinically? | Within a year | 1-2 years later | > 3 years later | Don't know | Still not used clinically |  |  |
| **16** | How would you rate your institution's experience and comfort level with clinical use of surface imaging? | Expert | Developing | Amateur | Inexperienced |  |  |  |
| **17** | At your institution, what do you use as the reference surface for initial patient positioning in your surface imaging system? (Select all that apply.) | External surface from planning CT (i.e., DICOM) | Surface acquired with SI cameras in simulation room | Surface acquired with SI cameras in treatment room | Depends on the treatment site | Depends on the patient | Don't know |  |
| **18** | How commonly is surface imaging used for initial patient positioning of the following treatments at your institution? | Routinely | Occasionally | Rarely | Never | Not applicable | Don't Know |  |
|  | Breast/Chest wall | ______ | ______ | ______ | ______ | ______ | ______ |  |
|  | Head and neck | ______ | ______ | ______ | ______ | ______ | ______ |  |
|  | Intracranial SRS | ______ | ______ | ______ | ______ | ______ | ______ |  |
|  | SBRT | ______ | ______ | ______ | ______ | ______ | ______ |  |
|  | Extremity | ______ | ______ | ______ | ______ | ______ | ______ |  |
|  | Lung | ______ | ______ | ______ | ______ | ______ | ______ |  |
|  | GU/Prostate | ______ | ______ | ______ | ______ | ______ | ______ |  |
|  | Pediatrics | ______ | ______ | ______ | ______ | ______ | ______ |  |
|  | Other | ______ | ______ | ______ | ______ | ______ | ______ |  |
| **19** | For the treatments using surface imaging, how often is positioning verified with another imaging modality at your institution? | Daily | Weekly | Never | Other | Don't Know |  |  |
|  | Breast/Chest wall | ______ | ______ | ______ | ______ | ______ |  |  |
|  | Head and neck | ______ | ______ | ______ | ______ | ______ |  |  |
|  | Intracranial SRS | ______ | ______ | ______ | ______ | ______ |  |  |
|  | SBRT | ______ | ______ | ______ | ______ | ______ |  |  |
|  | Extremity | ______ | ______ | ______ | ______ | ______ |  |  |
|  | Lung | ______ | ______ | ______ | ______ | ______ |  |  |
|  | GU/Prostate | ______ | ______ | ______ | ______ | ______ |  |  |
|  | Pediatrics | ______ | ______ | ______ | ______ | ______ |  |  |
|  | Other | ______ | ______ | ______ | ______ | ______ |  |  |
| **20** | For the treatments using surface imaging, how often do you reacquire the reference surface for initial patient positioning during treatments NOT requiring bolus? Select the most relevant option. | Daily | Weekly | Depends on the Patient | Never | Don't Know |  |  |
|  | Breast/Chest wall | ______ | ______ | ______ | ______ | ______ |  |  |
|  | Head and neck | ______ | ______ | ______ | ______ | ______ |  |  |
|  | Intracranial SRS | ______ | ______ | ______ | ______ | ______ |  |  |
|  | SBRT | ______ | ______ | ______ | ______ | ______ |  |  |
|  | Extremity | ______ | ______ | ______ | ______ | ______ |  |  |
|  | Lung | ______ | ______ | ______ | ______ | ______ |  |  |
|  | GU/Prostate | ______ | ______ | ______ | ______ | ______ |  |  |
| **21** | For the treatments using surface imaging, how often do you reacquire the reference surface during treatments requiring bolus? If bolus is not used for these treatments, select "Not Applicable". | Daily | Weekly | Depends on the Patient | Never | Don't Know |  |  |
|  | Breast/Chest wall | ______ | ______ | ______ | ______ | ______ |  |  |
|  | Head and neck | ______ | ______ | ______ | ______ | ______ |  |  |
|  | Extremity | ______ | ______ | ______ | ______ | ______ |  |  |
| **22** | At your institution, what do you predominantly use as the reference surface for intra-fraction monitoring in your surface imaging system? (Select all that apply.) | External surface from planning CT (i.e., DICOM) | Surface acquired with SI cameras in simulation room | Surface acquired with SI cameras in treatment room | Depends on the treatment site | Depends on the patient | Don't know |  |
| **23** | How commonly is surface imaging used for intra-fraction monitoring of the following treatments at your institution? Select "Not Applicable" if your center does not provide this treatment. | Routinely | Occasionally | Rarely | Never | Not applicable | Don't know |  |
|  | Breast/Chest wall | ______ | ______ | ______ | ______ | ______ | ______ |  |
|  | Head and neck | ______ | ______ | ______ | ______ | ______ | ______ |  |
|  | Intracranial SRS | ______ | ______ | ______ | ______ | ______ | ______ |  |
|  | SBRT | ______ | ______ | ______ | ______ | ______ | ______ |  |
|  | Extremity | ______ | ______ | ______ | ______ | ______ | ______ |  |
|  | Lung | ______ | ______ | ______ | ______ | ______ | ______ |  |
|  | GU/Prostate | ______ | ______ | ______ | ______ | ______ | ______ |  |
|  | Pediatrics | ______ | ______ | ______ | ______ | ______ | ______ |  |
|  | Other | ______ | ______ | ______ | ______ | ______ | ______ |  |
| **24** | Please indicate the treatments for which surface imaging is used to gate the beam when performing respiratory motion management techniques at your institution. If you do not perform these at your institution, please select "Not Applicable". If you EXCLUSIVELY use non-SI systems for respiratory motion management for a given treatment, please select "Non-SI System Used". | Breath Hold Amplitude Gating Only with SI | Phase-Based Gating Only with SI | Both Breath-hold Amplitude Gating and Phase-Based Gating with SI | Non-Surface Imaging System Used | Not applicable |  |  |
|  | Breast/Chest Wall | **______** | **______** | **______** | **______** | **______** |  |  |
|  | Lung (non-SBRT) | **______** | **______** | **______** | **______** | **______** |  |  |
|  | Abdomen (non-SBRT) | **______** | **______** | **______** | **______** | **______** |  |  |
|  | SBRT | **______** | **______** | **______** | **______** | **______** |  |  |
| **25** | If you use surface imaging for respiratory motion management, what do you use to perform additional routine verification of the alignment of internal anatomy? Select all that apply for each treatment site. | kV Imaging | MV Imaging | Fluoroscopy | CBCT/CT on rails | Other | Nothing |  |
|  | Breast/Chest Wall | **______** | **______** | **______** | **______** | **______** | **______** |  |
|  | Lung (non-SBRT) | **______** | **______** | **______** | **______** | **______** | **______** |  |
|  | Abdomen (non-SBRT) | **______** | **______** | **______** | **______** | **______** | **______** |  |
|  | SBRT | **______** | **______** | **______** | **______** | **______** | **______** |  |
